# Boolean network modeling of *β*-cell apoptosis and insulin resistance in type 2 diabetes mellitus

**DOI:** 10.1186/s12918-019-0692-0

**Published:** 2019-04-05

**Authors:** Pritha Dutta, Lichun Ma, Yusuf Ali, Peter M.A. Sloot, Jie Zheng

**Affiliations:** 1 Interdisciplinary Graduate School, Nanyang Technogical University, Singapore, Republic of Singapore; 2 Biomedical Informatics Lab, School of Computer Science and Engineering, Nanyang Technological University, Singapore, Republic of Singapore; 3 Lee Kong Chian School of Medicine, Nanyang Technogical University, Singapore, Republic of Singapore; 4 Complexity Institute, Nanyang Technogical University, Singapore, Republic of Singapore; 5 School of Information Science and Technology, ShanghaiTech University, Shanghai, China

**Keywords:** Boolean model, Type 2 diabetes mellitus, Insulin resistance, *β*-cell apoptosis

## Abstract

**Background:**

Major alteration in lifestyle of human population has promoted Type 2 diabetes mellitus (T2DM) to the level of an epidemic. This metabolic disorder is characterized by insulin resistance and pancreatic *β*-cell dysfunction and apoptosis, triggered by endoplasmic reticulum (ER) stress, oxidative stress and cytokines. Computational modeling is necessary to consolidate information from various sources in order to obtain a comprehensive understanding of the pathogenesis of T2DM and to investigate possible interventions by performing *in silico* simulations.

**Results:**

In this paper, we propose a Boolean network model integrating the insulin resistance pathway with pancreatic *β*-cell apoptosis pathway which are responsible for T2DM. The model has five input signals, i.e. ER stress, oxidative stress, tumor necrosis factor *α* (TNF *α*), Fas ligand (FasL), and interleukin-6 (IL-6). We performed dynamical simulations using random order asynchronous update and with different combinations of the input signals. From the results, we observed that the proposed model made predictions that closely resemble the expression levels of genes in T2DM as reported in the literature.

**Conclusion:**

The proposed model can make predictions about expression levels of genes in T2DM that are in concordance with literature. Although experimental validation of the model is beyond the scope of this study, the model can be useful for understanding the aetiology of T2DM and discovery of therapeutic intervention for this prevalent complex disease. The files of our model and results are available at https://github.com/JieZheng-ShanghaiTech/boolean-t2dm.

## Background

Type 2 diabetes mellitus (T2DM) is characterized by insulin resistance at its onset. Persistence of insulin resistance leads to pancreatic *β*-cell dysfunction and in extreme cases to *β*-cell apoptosis [1–3]. Insulin resistance increases the load on *β*-cells to produce more insulin in order to maintain blood glucose at normal levels. This homeostasis is maintained as long as *β*-cells can meet the increased insulin demand. However, persistence of excessive nutrients could lead to hyperglycemia, elevated free fatty acids (FFA), and inflammation, which severely impair *β*-cell functions, leading to insulin resistance and *β*-cell apoptosis.

The ER in the *β*-cells is responsible for the production and secretion of insulin. The increased demand for insulin synthesis in the presence of high glucose and FFA levels triggers the accumulation of misfolded proteins in the ER, causing ER stress and the consequent activation of the unfolded protein response (UPR). UPR initially attempts to mitigate ER stress by degrading misfolded proteins and preventing their further accumulation. However, when ER stress is not mitigated, UPR activates the apoptosis signals [4–6]. 78 kDa glucose regulated protein (GRP78) serves as a sensor of protein misfolding [7]. Under non-stressed conditions, GRP78 binds to three UPR initiator proteins, i.e. inositol requiring 1 (IRE1), PKR-like ER kinase (PERK), and activating transcription factor 6 (ATF6), and maintains them in the inactive state [8]. Under stressed conditions, GRP78 dissociates from these three proteins, causing their activation and initiation of UPR.

When ER stress can be resolved, the UPR assists *β* cells in their survival. However, when ER stress cannot be resolved the UPR activates the pro-apoptotic signals [9]. Hyperglycemia causes oxidative stress through the generation of reactive oxygen species (ROS) [10]. In the absence of an appropriate antioxidant response, the system experiences redox imbalance, leading to the activation of oxidative stress-sensitive signaling pathways. Cytokines, including FasL, TNF *α*, and IL-6, play important roles in the induction of *β*-cell apoptosis [11–15] as well as insulin resistance [16, 17]. Caspases serve as the final mediators of apoptosis. The upstream apoptosis initiator caspases 8 and 9 are activated on receiving death signal from the death-inducing signaling complex (DISC) and apoptosome respectively, which in turn activate the downstream apoptosis effector caspases 3, 6 and 7, which ultimately execute apoptosis [18].

Computational modeling is necessary to consolidate information from various sources, such as listed above, in order to obtain a comprehensive understanding of the pathogenesis of T2DM and investigate possible interventions by performing *in silico* simulations. A few dynamic models of insulin resistance in T2DM have been proposed recently. For instance, Brannmark et al. [19] proposed an ordinary differential equation (ODE) model of insulin signaling in T2DM. Rajan et al. proposed an ODE model to study the contribution of Forkhead box protein O1 (FOXO1) to insulin resistance in T2DM [20]. Another paper [21] presented an ODE model to simulate the development of insulin resistance by hyperglycemia, FFA, ROS, and inhibition of glucose transporter type 1 (GLUT-1) and glucose transporter type 4 (GLUT-4). However, there exists no model of *β*-cell apoptosis occurring in the T2DM condition. Also, there is no existing work that attempts to integrate the insulin resistance and *β*-cell apoptosis pathways in order to obtain a comprehensive understanding of the molecular mechanisms underlying T2DM. To discover potential therapeutic interventions for T2DM, it is essential to have a more comprehensive model for the mechanisms causing T2DM pathogenesis.

Therefore, we propose a Boolean network model integrating the insulin resistance pathway and *β*-cell apoptosis pathway for the purpose of obtaining deeper insights into the mechanisms of development and progression of T2DM. The aforementioned existing models are ODE models, whereas we constructed a Boolean network model. The reason behind this selection is that ODE models require detailed kinetic knowledge and time-series data for accurate parameter estimation. However, the size of our proposed network is relatively big (consisting of 72 nodes) and hence obtaining time-series expression data for all the genes would be expensive as well as time-consuming. Also, estimating the parameters of the ODE model with the time-series expression data of only a small subset of genes would result in erroneous parameter values. Furthermore, in a Boolean network model, gene expression is represented by either TRUE (1) or FALSE (0). By simplifying the gene expression levels into binary states, Boolean networks are feasible for simulating the behaviour of large regulatory networks in a qualitative way.

In a Boolean network model the state of each gene is represented by either 1 (TRUE), indicating the gene is highly expressed, or 0 (FALSE) when the gene is lowly expressed. An edge in a Boolean network can be either activating or inhibiting [22]. In this paper, we have used random asynchronous Boolean simulation [23, 24], which updates genes in a random order in each iteration. This random asynchronous update method is inspired by the stochastic nature of gene regulatory networks, where gene expression alteration occurs in a random order rather than simultaneously [24].

Due to the lack of experimental gene expression data, we validate our simulation results by comparing predicted patterns of gene expression levels with experimental observations reported in the literature. We also analyze the dynamical behaviors of the model by visualizing the state transition graphs under different combinations of input signals. Our results show that the simple Boolean network model can capture some qualitative trends of the genetic circuits regulating the cell fate decision of *β*-cells, and shed light on the causes and processes of dysfunctional insulin metabolism and loss of *β*-cell homeostasis that occur in T2DM.

## Methods

**Fig. 1:**
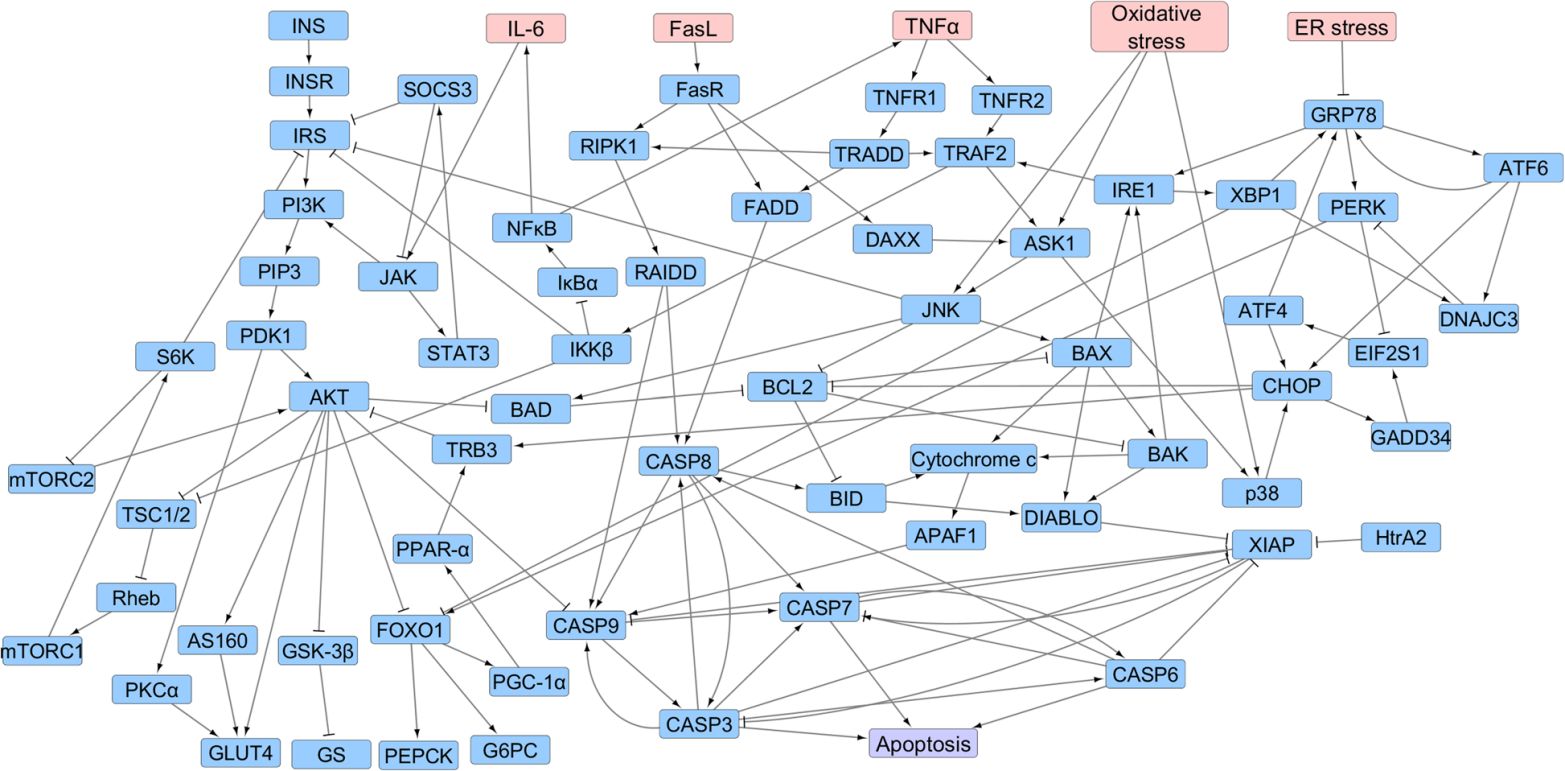
Gene Regulatory Network. Insulin resistance and *β*-cell apoptosis pathways involved in the pathogenesis of Type 2 diabetes mellitus. The red nodes denote the five input signals and the purple node represents *β*-cell apoptosis. A → B indicates activation of gene B by gene A, and A −| B indicates inhibition of gene B by gene A

In this paper, we propose a Boolean network model of *β*-cell fate in T2DM. The model was constructed by extracting information from the KEGG pathways [25] and literature. The gene interactions incorporated into the model with reference to the existing literature are listed in Table 1. In this model, we integrated the *β*-cell apoptosis pathway with the insulin resistance pathway, as shown in Fig. 1. The apoptosis pathway consists of the signaling pathways triggered by ER stress (UPR pathway), oxidative stress, and 3 cytokines, i.e. FasL, TNF *α*, and IL-6. The insulin resistance pathways consist of phosphatidylinositide 3-kinase (PI3K)-protein kinase B (PKB or AKT) (KEGG ID: hsa04151), mammalian target of rapamycin (mTOR) (KEGG ID: hsa04150), janus kinase (JAK)- signal transducer and activator of transcription (STAT) (KEGG ID: hsa04630), and insulin (KEGG ID: hsa04910) signaling pathways. T2DM first causes insulin resistance, i.e. insulin fails to bind to insulin receptors in cells, thereby blocking the uptake of blood glucose by cells. Sustained insulin resistance finally leads to *β*-cell failure and apoptosis.

**Table 1:** The gene interactions incorporated into the model with reference to the existing literature

Gene interations	Reference
IRE1 *↑*→ XBP1 *↑*→*β*-cell dysfunction	[26]
(IRE1 + TRAF2 + ASK1) *↑*→ JNK *↑*→ BCL2 (anti-apoptotic gene) *↓*	[28–30]
BCL2 *↓*→ (BAX + BAK) (pro-apoptotic) *↑*	[50, 51]
PERK *↑*→ EIF2S1 *↓*→ ATF4 *↑*→ CHOP (pro-apoptotic) *↑*	[27]
ATF6 *↑*→ CHOP (pro-apoptotic) *↑*→ BCL2 (anti-apoptotic gene) *↓*	[51, 52]
Oxidative stress *↑*→ ASK1 *↑*, JNK *↑*, p38 *↑*	[31–33]
p38 *↑*→ CHOP (pro-apoptotic) *↑*	[34]
FasL *↑*→ (FasR + FADD + pro-caspase-8) *↑*→ caspase-8 *↑*→ caspase-3 *↑*→ apoptosis	[53]
TNF *α↑*→ (TNFR1 + TRADD) *↑*→ RIPK1 *↑*, FADD *↑*, TRAF2 *↑*	[54]
FADD *↑*→ caspase-8 *↑*	[54]
RIPK1 *↑*→ RAIDD *↑*→ caspase-8 *↑*	[54]
TNF *α↑*→ TNFR2 TNF *α↑*→ TRAF2 *↑*→...→ JNK *↑*, NF-kB *↑*	[55–57]
(BAX + BAK) (pro-apoptotic) *↑*→ Cytochrome c *↑*→ (APAF1 + caspase-9) *↑*→ caspase-3 *↑*	[6, 58]
XIAP *↑*→ caspase-3 *↓*, caspase-7 *↓*, caspase-9 *↓*	[35, 36]
DIABLO *↑*, HtrA2 *↑*→ XIAP *↓*	[37]
INSR *↑*→ IRS *↑*→ PI3K *↑*→... → AKT *↑*→ FOXO1 *↓*, GSK3 *β↓*, GLUT4 *↑*	[59–61]
GSK3 *β↑*→ GS *↓*→ glycogen synthesis *↓*	[42, 43]
FOXO1 *↑*→ PEPCK *↑*, G6PC *↑*→ glucose synthesis *↑*	[47, 47–49]
(mTORC1 + S6K) *↑*→ IRS *↓*	[44–46]
IKK *β↑*→ TSC1/2 *↓*→ mTORC1 *↑*	[62]
ER stress *↑*→... → IRE1 *↑*→... → JNK *↑*→ IRS *↓*	[38, 39, 63]
ER stress *↑*→... → IRE1 *↑*→ XBP1 *↑*→ FOXO1 *↓*	[64]
PERK *↑*→ FOXO1 *↑*	[65]
ER stress *↑*→... → ATF4 *↑*→ CHOP *↑*→ TRB3 *↑*→ AKT *↓*	[40, 41]
IL-6 *↑*→ JAK *↑*→ STAT3 *↑*→ SOCS3 *↑*→ IRS *↓*	[66–68]

The Boolean update functions, listed in Table 2, for the target genes in the model are defined by combining activating input genes using OR functions and inhibiting input genes using AND functions. The reason behind this combination strategy is that a target gene will be expressed when at least one of its activating genes is expressed and all of its inhibiting genes are absent.

**Table 2:** Boolean functions for the Boolean model

Node	Boolean function	Node	Boolean function
ER	ER	OS	OS
FasL	FasL	TNF *α*	TNF *α* or NFKB
IL-6	IL-6 or NFKB		
GRP78	ATF6 or XBP1 or ATF4 and (not ER)	ATF6	GRP78
PERK	GRP78 and (not DNAJC3)	IRE1	BAX or BAK or GRP78
EIF2S1	GADD34 and (not PERK)	DNAJC3	ATF6 or XBP1
ATF4	EIF2S1	CHOP	ATF6 or ATF4
XBP1	IRE1	GADD34	CHOP
TNFR1	TNF *α*	TNFR2	TNF *α*
TRAF2	IRE1 or TNFR2 or TRADD	ASK1	OS or TRAF2 or DAXX
JNK	OS or ASK1 or GADD45	p38	OS or ASK1
BCL2	(not JNK) and (not CHOP) and (not P53) and (not BAD)	BID	CASP8 and (not BCL2)
BAX	JNK or P53 and (not BCL2)	BAK	BAX and (not BCL2)
DIABLO	BAX or BAK or BID	HtrA2	BAX or BAK or BID
FasR	FasL	TRADD	TNFR1
DAXX	FasR	RIPK1	FasR or TRADD
RAIDD	RIPK1	FADD	FasR or TRADD
CASP8	RAIDD or FADD or CASP3 or CASP6	CASP9	RAIDD or CASP8 or CASP3 or APAF1 or CASP12 and (not XIAP) and (not AKT)
CASP3	CASP9 or CASP8 and (not XIAP)	CASP7	CASP9 or CASP8 or CASP3 or CASP6 and (not XIAP)
CASP6	CASP7 or CASP3		
XIAP	(not DIABLO) and (not HtrA2) (not CASP3)	CytochromeC	BAX or BAK or BID
APAF1	CytochromeC or P53	Apoptosis	CASP3 or CASP6 or CASP7
INS	INS	INSR	INS
IRS	INSR and (not SOCS3) and (not JNK) (not IKK *β*) and (not S6K)	PI3K	IRS or JAK
PIP3	PI3K	PDK1	PIP3
AKT	PDK1 or mTORC2 and (not TRB3)	AS160	AKT
PKC *α*	PDK1	GLUT4	AKT or AS160 or PKC *α*
GSK3 *β*	not AKT	GS	not GSK3 *β*
FOXO1	PERK and (not AKT) and (not XBP1)	PGC1 *α*	FOXO1
PEPCK	FOXO1	G6PC	FOXO1
PPAR *α*	PGC1 *α*	TRB3	PPAR *α* or CHOP
TSC1/2	(not AKT) and (not IKK *β*)	Rheb	not TSC1/2
mTORC1	Rheb	S6K	mTORC1
mTORC2	not S6K	BAD	JNK and (not AKT)
JAK	IL-6 and (not SOCS3)	STAT3	JAK
SOCS3	STAT3	IKK *β*	TRAF2
NF *κ*B	not IKB *α*	IKB *α*	not IKK *β*

The proposed Boolean network consists of 72 nodes, of which five are input signals, one node represents Apoptosis, and the remaining 66 nodes represent genes. We employ the random asynchronous Boolean update [23, 24] method to perform the simulations. The random asynchronous Boolean method first generates a random permutation of the nodes at each time step and updates the states of the nodes in the order specified by the permutation. This allows us to capture the stochastic changes in gene expressions that occur in real gene regulatory networks. The random asynchronous Boolean simulations were performed using the Python code provided in [23] which is available at https://gitlab.com/stemcellbioengineering/garuda-boolean.

For example, suppose a gene regulatory network consists of 3 genes, {*g*_1_,*g*_2_,*g*_3_}. The Boolean update functions for the genes are as follows:

$$g_{1} = g_{3} $$

$$g_{2} = g_{1} \lor g_{3} $$

$$g_{3} = g_{2} $$

Suppose an iteration randomly generates a permutation of nodes as {3,1,2}. Then the asynchronous Boolean updates will be carried out as follows:

$$g_{3}(t+1) = g_{2}(t) $$

$$g_{1}(t+1) = g_{3}(t+1) $$

$$g_{2}(t+1) = g_{1}(t+1) \lor g_{3}(t+1) $$

From the above equations, we see that the nodes are updated in a randomly generated order as specified by the permutation, rather than simultaneously.

**Fig. 2:**
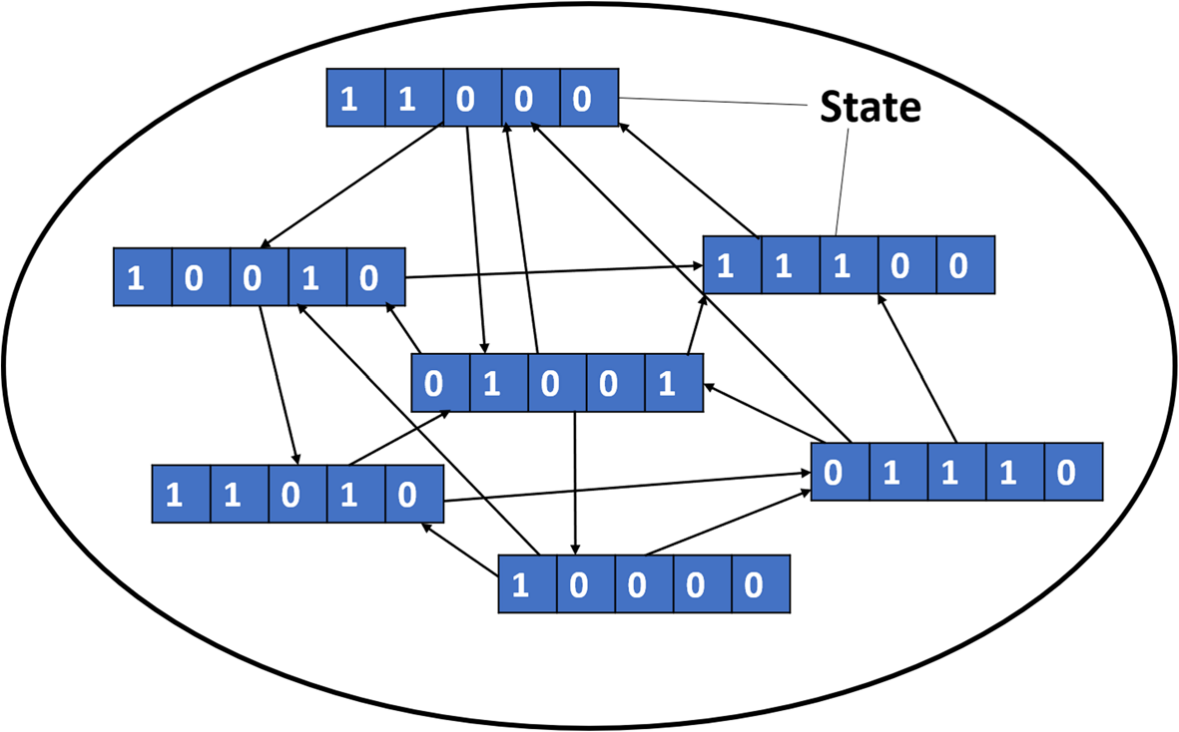
Strongly Connected Component. An example of a strongly connected component (SCC). Suppose the network consists of five genes. Then each node is a state which contains the expression levels of the five genes. An arrow from state *S*_1_ to state *S*_2_ indicates an update step. In an SCC all states can be reached from every other state

After performing the simulations for a fixed number of iterations, a directed graph of states is obtained, where each state is a vector representing the expression levels of all genes at a particular time step. The strategy of strongly connected components (SCCs) is employed on this directed graph to capture the dynamic nature of the states [23]. An SCC of a directed graph is a sub-graph that is strongly connected, i.e., each node is reachable from every other node in the sub-graph. An illustration of SCC is given in Fig. 2. Each node is a state with the expression levels of all the genes in the network (for the example we assume a network with five genes) and there is a path between each pair of nodes in both directions. Let us consider that an SCC consists of a set of *N* states {*S*_1_,*S*_2_,...,*S*_*N*_}. The probability of state *S*_*i*_ being one of the states of the SCC is given by:

$$P(S_{i}) = \frac{\text{ number of occurrences of } S_{i}}{\sum_{j=1}^{N}\text{ number of occurrences of } S_{j}}. $$

We calculate the gene expression level of each gene in a particular SCC as the sum of probabilities of states where the gene is in the ON state. Therefore, the expression level of a gene, *g*_*i*_, with respect to an SCC is determined as follows:

$$Exp(g_{i}) = \sum_{S_{j} \in OnSt(g_{i})}P(S_{j}) $$

where

$$OnSt(g_{i}) = \{S_{j} \in SCC \mid g_{i}(S_{j})=1\}. $$

It is easy to see that

$$\sum_{j=1}^{N}P(S_{j}) = 1 $$

We use ER stress, oxidative stress, TNF *α*, FasL, and IL-6 as input signals. Also, based on the literature, some of the nodes are assigned specific values (Table 3) and the rest are set to random values as initial conditions. We performed simulations using different combinations of the input signals, as shown in Table 4. We carried out 1000 simulation runs and 1000 Boolean update steps per simulation for each input signal. The results of the simulations are presented and discussed in the following section.

**Table 3:** Initial conditions

Node	Initial value	Reason
Apoptosis	False	We set apoptosis to False to see whether the input signals can cause apoptosis
Caspases 3, 6, 7, 8, 9	False	Caspases serve as the final mediators of apoptosis. So, we set them to False to see whether the input signals can activate them

**Table 4:** Different combinations for the input signal nodes

	ER stress	Oxidative stress	TNF *α*	FasL	IL-6
Case 1	True	False	False	False	False
Case 2	False	True	False	False	False
Case 3	True	True	False	False	False
Case 4	False	False	True	False	False
Case 5	False	False	False	True	False
Case 6	False	False	False	False	True
Case 7	False	False	True	True	True
Case 8	True	True	True	True	True

Due to the lack of experimental data, we validate our proposed Boolean network model using relevant literature (see Table 1). For each gene *g*_*i*_, we use the same symbol *g*_*i*_ to represent its binary expression level.

$$ g_{i} \,=\, \begin{cases} 1 & \text{if }g_{i} \text{ is reported as expressed in the literature} \\ 0 & \text{if }g_{i} \text{ is reported as not expressed in the literature} \end{cases} $$

In our model, we determine the expression level of each gene with respect to a particular SCC. Thus the gene expression levels are in the range [0, 1]. We assume that if the expression value of a gene is greater than 0.50, then the gene is expressed, otherwise, it is not expressed.

For the purpose of validating our proposed model, we employ the performance metrics of precision, recall (sensitivity), specificity, and F1 score. The simulation result of our proposed model is verified against the literature as follows. For each gene *g*_*i*_,

$$ g_{i} \in \begin{cases} \text{True positive,} & \text{if }g_{i} = 1 \text{ (simulation result) and }g_{i} = 1 \text{ (literature)} \\ \text{True negative,} & \text{if }g_{i} = 0 \text{ (simulation result) and }g_{i} = 0 \text{ (literature)} \\ \text{False positive,} & \text{if }g_{i} = 1 \text{ (simulation result) and }g_{i} = 0 \text{ (literature)} \\ \text{False negative,} & \text{if }g_{i} = 0 \text{ (simulation result) and }g_{i} = 1 \text{ (literature)} \end{cases} $$

The four evaluation metrics are calculated using the following formulae:

$$Precision = \frac{\textit{True positive}}{\textit{True positive} + \textit{False positive}} $$

$$Recall or sensitivity = \frac{\textit{True positive}}{\textit{True positive} + \textit{False negative}} $$

$$Specificity = \frac{\textit{True negative}}{\textit{True negative} + \textit{False positive}} $$

$$F1\ \ {score} = \frac{2 \times precision \times recall}{precision + recall} $$

## Results

### Comparison with the literature

The expression levels of genes in the SCCs obtained by performing simulations with our proposed Boolean model are listed in Tables 5 and 6. Simulations performed using input signal cases 1, 2, 3, 4, 5, 7, and 8 (Table 4) result in two attractors (SCCs). Apoptosis is ON in both of the attractors. Simulations performed using input signal case 6 (Table 4) result in six attractors (SCCs). Apoptosis is ON in four attractors and OFF in the remaining two attractors. These observations are consistent with the literature where ER stress, oxidative stress, and cytokines have been shown to cause apoptosis of *β*-cells individually as well as together [4–6].

**Table 5:** Gene expressions of the significant genes in the model for input signal cases 1-5 and 7-8

Node	Case 1		Case 2		Case 3		Case 4		Case 5		Case 7		Case 8	
	A1	A2	A1	A2	A1	A2	A1	A2	A1	A2	A1	A2	A1	A2
Apoptosis	1	1	1	1	1	1	1	1	1	1	1	1	1	1
AKT	0.50	0.50	0.49	0.49	0.49	0.49	0.49	0.49	0.50	0.49	0.49	0.50	0.50	0.49
APAF-1	1	1	1	1	1	1	1	1	1	1	1	1	1	1
ASK1	1	1	1	1	1	1	1	1	1	1	1	1	1	1
ATF4	1	0	1	0	1	0	1	0	1	0	1	0	1	0
ATF6	0	0	0	0	0	0	0	0	0	0	0	0	0	0
BAK	1	1	1	1	1	1	1	1	1	1	1	1	1	1
BAX	1	1	1	1	1	1	1	1	1	1	1	1	1	1
BCL2	0	0	0	0	0	0	0	0	0	0	0	0	0	0
Caspase-3	1	1	1	1	1	1	1	1	1	1	1	1	1	1
Caspase-6	1	1	1	1	1	1	1	1	1	1	1	1	1	1
Caspase-7	1	1	1	1	1	1	1	1	1	1	1	1	1	1
Caspase-8	1	1	1	1	1	1	1	1	1	1	1	1	1	1
Caspase-9	1	1	1	1	1	1	1	1	1	1	1	1	1	1
CHOP	1	0	1	0	1	0	1	0	1	0	1	0	1	0
DIABLO	1	1	1	1	1	1	1	1	1	1	1	1	1	1
EIF2S1	1	0	1	0	1	0	1	0	1	0	1	0	1	0
FADD	1	1	1	1	1	1	1	1	1	1	1	1	1	1
FASR	0	0	0	0	0	0	0	0	0	0	0	0	0	0
FOXO1	0	0	0	0	0	0	0	0	0	0	0	0	0	0
G6PC	0	0	0	0	0	0	0	0	0	0	0	0	0	0
GADD34	1	0	1	0	1	0	1	0	1	0	1	0	1	0
GLUT4	0.65	0.65	0.65	0.65	0.65	0.65	0.65	0.65	0.65	0.65	0.65	0.65	0.65	0.65
GRP78	1	1	1	1	1	1	1	1	1	1	1	1	1	1
GS	0.50	0.50	0.50	0.49	0.49	0.49	0.49	0.49	0.50	0.50	0.50	0.50	0.50	0.49
GSK3 *β*	0.49	0.49	0.50	0.50	0.50	0.50	0.50	0.50	0.49	0.49	0.49	0.49	0.49	0.50
HtrA2	1	1	1	1	1	1	1	1	1	1	1	1	1	1
IKB *α*	0	0	0	0	0	0	0	0	0	0	0	0	0	0
IKK *β*	1	1	1	1	1	1	1	1	1	1	1	1	1	1
INS	1	1	1	1	1	1	1	1	1	1	1	1	1	1
INSR	1	1	1	1	1	1	1	1	1	1	1	1	1	1
IRE1	1	1	1	1	1	1	1	1	1	1	1	1	1	1
IRS	0	0	0	0	0	0	0	0	0	0	0	0	0	0
JAK	0.50	0.49	0.50	0.49	0.49	0.50	0.49	0.49	0.49	0.49	0.49	0.50	0.50	0.50
JNK	1	1	1	1	1	1	1	1	1	1	1	1	1	1
NFKB	1	1	1	1	1	1	1	1	1	1	1	1	1	1
PEPCK	0	0	0	0	0	0	0	0	0	0	0	0	0	0
PERK	0	0	0	0	0	0	0	0	0	0	0	0	0	0
PI3K	0.50	0.49	0.49	0.49	0.50	0.50	0.50	0.50	0.50	0.49	0.49	0.50	0.50	0.49
RAIDD	1	1	1	1	1	1	1	1	1	1	1	1	1	1
RIPK1	1	1	1	1	1	1	1	1	1	1	1	1	1	1
S6K	1	1	1	1	1	1	1	1	1	1	1	1	1	1
SOCS3	0.49	0.49	0.50	0.49	0.50	0.50	0.49	0.49	0.50	0.50	0.49	0.49	0.49	0.50
STAT3	0.50	0.49	0.49	0.49	0.49	0.50	0.49	0.49	0.50	0.50	0.49	0.49	0.50	0.50
TNFR1	1	1	1	1	1	1	1	1	1	1	1	1	1	1
TNFR2	1	1	1	1	1	1	1	1	1	1	1	1	1	1
TRADD	1	1	1	1	1	1	1	1	1	1	1	1	1	1
TRAF2	1	1	1	1	1	1	1	1	1	1	1	1	1	1
TRB3	1	0	1	0	1	0	1	0	1	0	1	0	1	0
TSC2	0	0	0	0	0	0	0	0	0	0	0	0	0	0
XBP1	1	1	1	1	1	1	1	1	1	1	1	1	1	1
XIAP	0	0	0	0	0	0	0	0	0	0	0	0	0	0
mTORC1	1	1	1	1	1	1	1	1	1	1	1	1	1	1
p38	1	1	1	1	1	1	1	1	1	1	1	1	1	1

Here A1 and A2 denotes SCC1 and SCC2

**Table 6:** Gene expressions of the significant genes in the model for input signal case 6. Here A1-A6 denotes SCC1-SCC6

Node	Case 6					
	A1	A2	A3	A4	A5	A6
Apoptosis	1	1	1	1	0	0
AKT	0.49	0.49	0.63	0.55	0.65	0.56
APAF-1	1	1	0	1	0	0
ASK1	1	1	0	0	0	0
ATF4	1	0	0	1	0	1
ATF6	0	0	0	0	0	0
BAK	1	1	0	0	0	0
BAX	1	1	0	0	0	0
BCL2	0	0	1	0	1	0
Caspase-3	1	1	1	1	0	0
Caspase-6	1	1	1	1	0	0
Caspase-7	1	1	1	1	0	0
Caspase-8	1	1	1	1	0	0
Caspase-9	1	1	1	1	0	0
CHOP	1	0	0	1	0	1
DIABLO	1	1	0	1	0	0
EIF2S1	1	0	0	1	0	1
FADD	1	1	0	0	0	0
FASR	0	0	0	0	0	0
FOXO1	0	0	0	0	0	0
G6PC	0	0	0	0	0	0
GADD34	1	0	0	1	0	1
GLUT4	0.65	0.65	0.75	0.70	0.78	0.71
GRP78	1	1	0	1	0	1
GS	0.49	0.49	0.62	0.54	0.65	0.55
GSK3 *β*	0.50	0.50	0.38	0.45	0.36	0.45
HtrA2	1	1	0	1	0	0
IKB *α*	0	0	1	1	1	1
IKK *β*	1	1	0	0	0	0
INS	1	1	1	1	1	1
INSR	1	1	1	1	1	1
IRE1	1	1	0	0	0	0
IRS	0	0	0.19	0.22	0.18	0.22
JAK	0.49	0.49	0.49	0.49	0.49	0.50
JNK	1	1	0	0	0	0
NFKB	1	1	0	0	0	0
PEPCK	0	0	0	0	0	0
PERK	0	0	0	0	0	0
PI3K	0.49	0.49	0.54	0.55	0.54	0.56
RAIDD	1	1	0	0	0	0
RIPK1	1	1	0	0	0	0
S6K	1	1	0.62	0.55	0.62	0.56
SOCS3	0.49	0.50	0.50	0.49	0.49	0.49
STAT3	0.49	0.49	0.49	0.49	0.49	0.50
TNFR1	1	1	0	0	0	0
TNFR2	1	1	0	0	0	0
TRADD	1	1	0	0	0	0
TRAF2	1	1	0	0	0	0
TRB3	1	0	0	1	0	1
TSC2	0	0	0.37	0.45	0.36	0.44
XBP1	1	1	0	0	0	0
XIAP	0	0	0	0	1	1
mTORC1	1	1	0.63	0.55	0.63	0.57
p38	1	1	0	0	0	0

From our simulation results, we observe that Caspases 3, 6, 7, 8, and 9, which serve as the final mediators of apoptosis [18] are TRUE in the attractors, even though in the initial condition they were set to FALSE. The ER stress sensor IRE1 and its downstream gene X-box protein binding 1 (XBP1) are TRUE in some attractors, and FALSE in others [26]. Another ER stress sensor, PERK is observed to be FALSE in all the attractors. Also, eukaryotic translation initiation factor 2 subunit 1 (EIF2S1), activating transcription factor 4 (ATF4), and C/EBP homologous protein (CHOP) are TRUE in some attractors and FALSE in the others. PERK phosphorylates and inactivates EIF2S1, which inhibits protein synthesis. Phosphorylated EIF2S1 increases the translation of ATF4 [8], which in turn activates pro-apoptotic CHOP, causing *β*-cell dysfunction and death [27]. The attractors where IRE1, XBP1, EIF2S1, ATF4, and CHOP have expression levels of 0 may denote the transition states when these genes are not contributing to apoptosis.

While associating with TNF-receptor-associated factor 2 (TRAF2) and apoptosis signal-regulating kinase 1 (ASK1), IRE1 activates jun N-terminal kinase (JNK) [28, 29], which in turn inhibits the anti-apoptotic protein B-cell lymphoma 2 (BCL2) [30]. Oxidative stress activates ASK1 [31, 32], JNK and p38 [33]. Activated p38 phosphorylates and elevates the expression of pro-apoptotic CHOP [34]. From the simulation results, we observe that the pro-apoptotic genes, TRAF2, ASK1, JNK, p38, BAX, and BAK are TRUE and the anti-apoptotic gene BCL2 is FALSE in one attractor, while the reverse states are observed in the other. X-linked inhibitor of apoptosis protein (XIAP), which inhibits Caspases 3, 7, and 9 [35, 36], has an expression level of 0, whereas direct IAP-binding protein with low pI (DIABLO) and high temperature requirement protein A2 (HtrA2), which inhibit XIAP [37], have expression levels of 1.

JNK phosphorylates and inhibits insulin receptor substrate (IRS) [38, 39]. IRS gene is FALSE in both of the attractors. PI3K has an expression level of around 0.50 in all the attractors. Tribbles homolog 3 (TRB3) is induced by ER stress through the ATF4-CHOP pathway [40]. Over-expression of TRB3 inhibits AKT and decreases glucose uptake [41]. TRB3 is TRUE in one attractor and FALSE in the other. AKT has an expression level of 0.50 in both of the attractors. Thus, from the results, we observe that ER stress inhibits the PI3K-AKT signaling pathway and promotes insulin resistance.

Insulin promotes conversion of glucose to glycogen by inhibiting glycogen synthase kinase-3 *β* (GSK3 *β*) through the PI3K-AKT signaling pathway, which leads to the activation of glycogen synthase (GS) [42]. From the simulation results, we observe that the expression level of GSK3 *β*, which inhibits glycogen synthesis through inhibition of GS [42, 43] is approximately 0.49 and that of GS is approximately 0.50. From these simulation results, we can infer that glycogen synthesis is reduced which contributes to insulin resistance.

In T2DM, the mammalian target of rapamycin complex 1 (mTORC1)/ S6 kinase (S6K) signaling is activated [44] leading to the inhibition of IRS [45, 46]. We observe from the simulation results that mTORC1 and S6K have expression levels of 1 thus inhibiting IRS which has an expression of 0. These events cause PI3K and AKT to have low expression levels of approximately 0.50, which in turn reduces glucose uptake through GLUT4 whose expression level is around 0.65.

FOXO1 increases the expression of phosphoenolpyruvate carboxykinase (PEPCK) and glucose-6-phosphatase (G6PC) and thus promotes glucose synthesis [47]. Insulin inhibits the expression of FOXO1 through the activation of the PI3K/AKT signaling pathway, which in turn suppresses PEPCK and G6PC, and thereby reduces glucose synthesis [47–49]. From our simulation results, we observe that FOXO1, PEPCK, and G6PC are FALSE. This could be due to the fact that PI3K and AKT are not completely inactive, though they may have low expression levels, and hence is still able to inhibit the expressions of FOXO1, PEPCK, and G6PC.

In Case 6 where only signal IL6 is active, we observe six attractors (Table 6), of which four indicate apoptosis and two do not. For the attractors where apoptosis is observed, the expression levels of the genes are similar to those mentioned above for the other input signal cases. When apoptosis is not observed, i.e. in the two remaining attractors, the caspases, JNK, BAX, and BAK are FALSE. In one of these two attractors, BCL2 is FALSE and CHOP is TRUE. In the other attractor we observe the reverse expression pattern. Thus, in the presence of only IL-6, apoptosis may or may not be activated.

We further assessed the performance of our proposed Boolean network model by comparing model predictions of gene expressions against the literature. Considering the simulation results obtained using the 8 input signals listed in Table 4, the average precision, recall (sensitivity), specificity, and F1 score obtained for our model are 0.9524, 0.8, 0.875, and 0.8696, respectively. We observe that the validation scores for our model are not very high, maybe because our model is sensitive to some missing interactions.

### State transition graphs

**Fig. 3:**
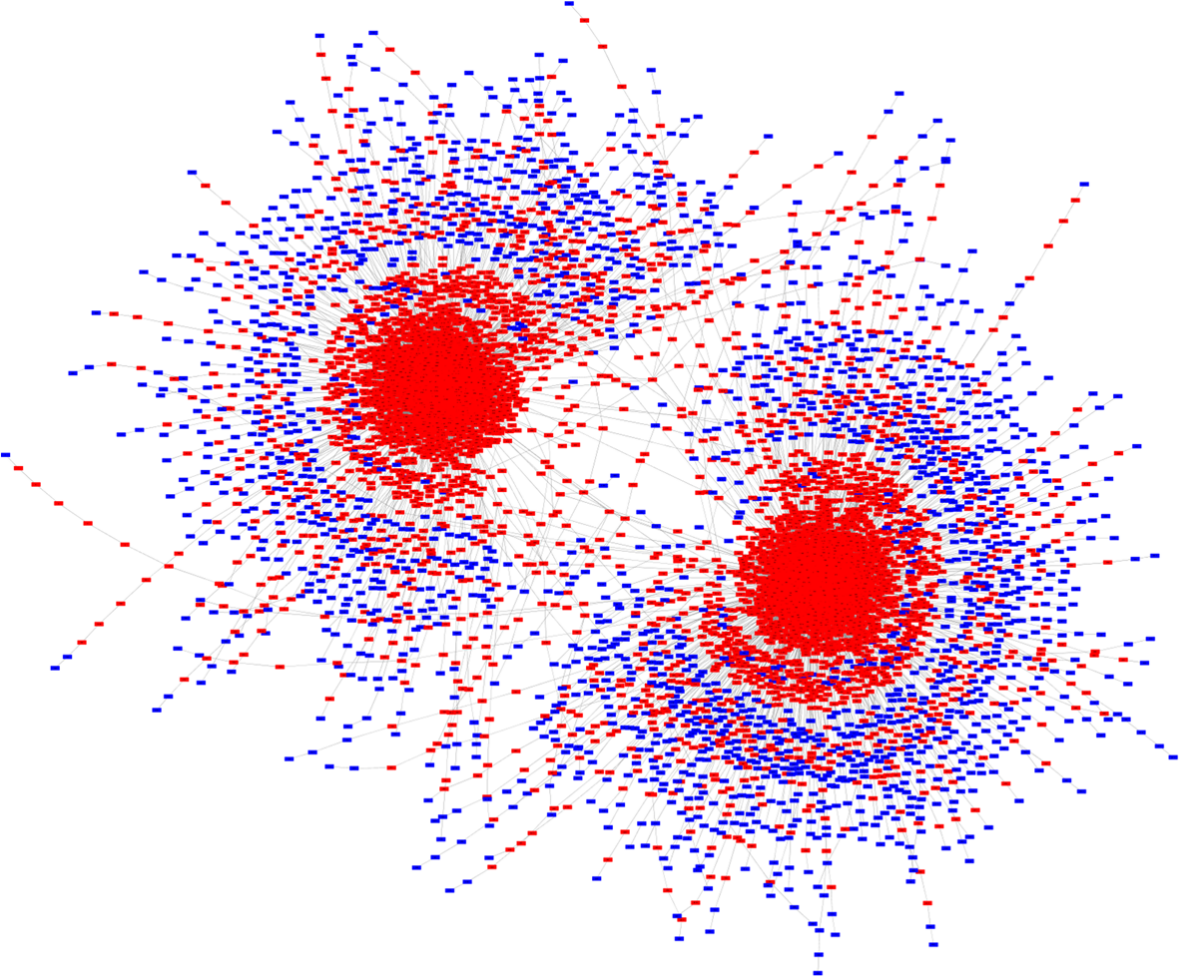
State Transition Graph 1. State transition graph obtained by simulating our proposed Boolean network model using input signal condition given in Case 8 of Table 4. Simulations generate 2 attractors, both having the Apoptosis node activated. Apoptosis is ON in the red coloured states and OFF in the blue colored states

Figure 3 shows the state transition graph of the state space generated by simulations conducted using input signal combination given in case 8 (Table 4). The two dense red regions represent the two SCCs where apoptosis is ON. The blue nodes represent states where apoptosis is OFF. Thus from the state transition graph, we observe that, in the presence of all input signals, apoptosis is eventually activated, even though in the initial condition it is set to FALSE.

**Fig. 4:**
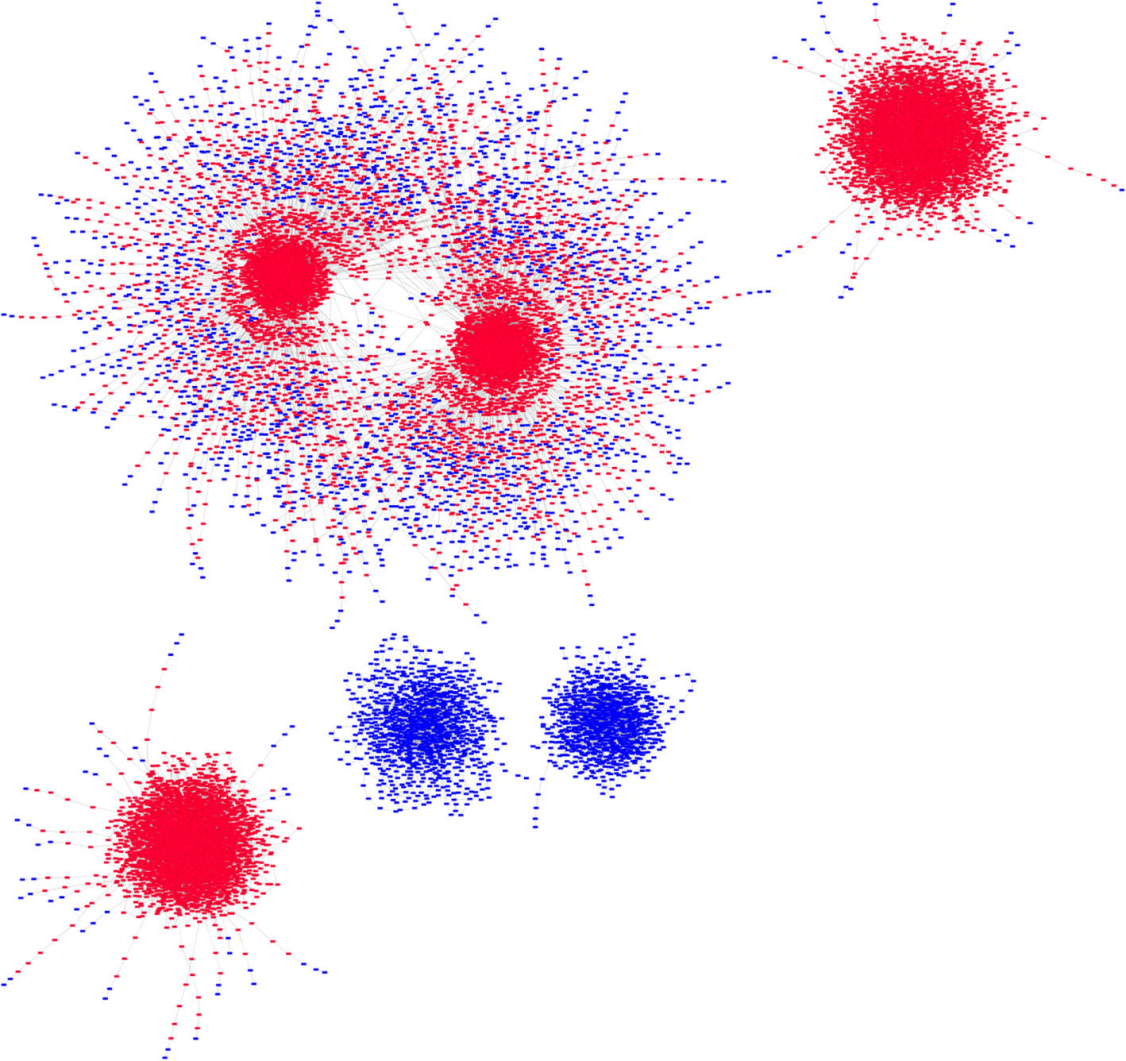
State Transition Graph 2. State transition graph obtained by simulating our proposed Boolean network model using input signal condition given in Case 6 of Table 4. Simulations generate 6 attractors. In four of the attractors Apoptosis is ON, denoted by red colour, and in the remaining two attractors Apoptosis is OFF, denoted by blue colour

Figure 4 shows the state transition graph of the state space generated by simulations conducted using input signal combination given in case 6 (Table 4). The four dense red regions represent the four SCCs where apoptosis is ON. The two dense blue regions represent the two SCCs where apoptosis is OFF. Thus from the state transition graph, we observe that, in the presence of only IL-6, apoptosis may or may not be activated.

### Comparison with random Boolean networks

We also compared our Boolean network model with random Boolean network models using the 8 input signal combinations given in Table 4. For cases 1, 2, 3, 4, 5, 7, and 8 we found that the number of attractors obtained by simulating the random Boolean networks ranges from 28 to 177, whereas for our Boolean network model the number of attractors is 2. Similarly, for case 6, the number of attractors obtained by simulating the random Boolean networks ranges from 25 to 180, whereas for our Boolean network model the number of attractors is 6. Thus, from the results we observe that the random Boolean networks typically have large numbers of attractors.

## Conclusion

In this paper, we proposed a Boolean network model of the integrated insulin resistance and *β*-cell apoptosis pathways. Such a model, which explores the combined mechanism and interplay between insulin resistance and *β*-cell apoptosis in the pathogenesis of T2DM, has not been proposed before. We used the model to simulate the dynamics of gene expression induced by different combinations of the five input signals, i.e. ER stress, oxidative stress, and cytokines (TNF *α*, FasL, IL-6), which serve as triggers for insulin resistance and *β*-cell apoptosis.

The random order asynchronous update method was employed to perform the simulations, i.e. all nodes were updated in a random order at each update step. We assessed the performance of our model using the metrics of precision, recall (sensitivity), specificity, and F1 score, when validating our model against the literature. The precision score obtained is high, but sensitivity, specificity, and F1 scores are not so. One possible reason may be that some missing interactions affect the predictions of our model. We also compared our Boolean network model with random Boolean network models and observed that random Boolean networks typically have large numbers of attractors ranging from around 25 to 180, whereas our model shows small numbers of attractors ranging from 2 to 6.

As a future step, we can use this model to perform virtual gene knockout experiments to determine genes that play pivotal roles in insulin resistance and/or *β*-cell apoptosis, and these genes could be further investigated for possible disease interventions.

## Abbreviations

AKT (PKB): Protein kinase B
APAF1: Apoptotic protease-activating factor 1
ASK1: Apoptosis signal-regulating kinase 1
ATF4: Activating transcription factor 4
ATF6: Activating Transcription Factor 6
BCL2: B-cell lymphoma 2
CHOP: C/EBP homologous protein
DIABLO: Direct IAP-binding protein with low pI
EIF2S1: Eukaryotic translation initiation factor 2 subunit 1
ER: Endoplasmic reticulum
FADD: Fas-associated death domain-containing protein
FasL: Fas ligand
FasR: Fas receptor
FFA: Free fatty acids
FOXO1: Forkhead box protein O1
GADD34: Growth arrest and DNA damage-inducible protein
G6PC: Glucose-6-phosphatase
GLUT-1: Glucose transporter type 1
GLUT-4: Glucose transporter type 4
GRP78: 78 kDa glucose regulated protein
GS: Glycogen synthase
GSK3 *β*: Glycogen synthase kinase-3 *β*
HtrA2: High temperature requirement protein A2
IL-6: Interleukin-6
INSR: Insulin receptor
IRE1: Inositol Requiring 1
IRS: Insulin receptor substrate
JAK: Janus kinase
JNK: Jun N-terminal kinase
mTORC1: Mammalian target of rapamycin complex 1
mTORC2: Mammalian target of rapamycin complex 2
ODE: Ordinary differential equation
PEPCK: Phosphoenolpyruvate carboxykinase
PERK: PKR-like ER kinase
PI3K: Phosphatidylinositide 3-kinase
Rheb: Ras homolog enriched in brain
RIPK1: Receptor-interacting serine/threonine-protein kinase 1
ROS: Reactive oxygen species
S6K: S6 kinase
SCC: Strongly connected component
SOCS3: Suppressor of cytokine signaling 3
STAT3: Signal transducer and activator of transcription 3
T2DM: Type 2 Diabetes Mellitus
TNF *α*: Tumor necrosis factor *α*
TNFR1: Tumor necrosis factor receptor superfamily member 1A
TNFR2: Tumor necrosis factor receptor superfamily member 1B
TRADD: TNFR1-associated death domain
TRAF2: TNF-receptor-associated factor 2
TRB3: Tribbles homolog 3
TSC: Tuberous sclerosis complex
UPR: Unfolded pro-tein respons
XBP1: X-box protein binding 1
XIAP: X-linked inhibitor of apoptosis protein
